# Activated Lymphocyte-Based Immunotherapy Plus Tomotherapy in an Older Patient with Stage III Lung Cancer: A Case Report

**DOI:** 10.3390/geriatrics11010014

**Published:** 2026-01-29

**Authors:** Anastasia Ganina, Madina Karimova, Dana Idrissova, Aigul Brimova, Manarbek Askarov, Larissa Kozina

**Affiliations:** 1 JSC National Scientific Medical Center, 42 Abilay Khan Ave., Astana 010009, Kazakhstan; madina.karimova.97@bk.ru (M.K.); illak@mail.ru (M.A.); l.kozina@nnmc.kz (L.K.); 2 International Oncological Tomotherapy Center “YMIT”, 42/1 Abilay Khan Ave., Astana 010009, Kazakhstan; didrissova888@gmail.com (D.I.); aigul_umit@mail.ru (A.B.)

**Keywords:** lung cancer, immunotherapy, activated lymphocytes, circulating tumor cells

## Abstract

Lung cancer is one of the most common malignant tumors and is associated with a high mortality rate, especially in aged patients. Immunotherapy is an effective method for treating lung cancer, particularly when used in combination with other treatments like chemotherapy. One of the types of immunotherapy is the use of autologous immune cells that are pre-activated before injection back to a patient. The effectiveness of this type of immunotherapy is determined by the specificity of its action on cancer cells through the activation of immune cell, e.g., lymphocytes. However, this treatment is not extensively used in elder patients due to higher risk of complications. On the other hand, in those aged patients who suffer from late stage cancer, the immune-cell based immunotherapy may come as a last resort. In this study, we present a clinical case of a 63-year-old patient with advanced-stage lung cancer and CT-confirmed infiltration of the left main bronchus. Treatment of the patient with immunotherapy using autologous activated lymphocytes combined with tomotherapy resulted in prominent improvement and decreased size of the malignancy. This positive effect was accompanied by a decrease in the number of circulating tumor cells in the blood. The patient was treated in May-June 2024 and is still alive with good condition as of August 2025. We conclude that combined treatment is a reliable option for selected aged patients with advanced-stage lung cancer.

## 1. Introduction

Lung cancer, the most common malignant tumor and deadliest cancer in the world [1,2], is characterized by a latent course and early appearance of metastases. It is classified into small cell lung cancer (SCLC) and non-small cell lung cancer (NSCLC). NSCLC is diagnosed in 80–85% of cases and subtyped into adenocarcinomas, squamous cell cancers, and large cell lung cancers [3,4]. Despite new treatment methods, NSCLC is characterized by a low survival rate, ranging from 12 to 15%. Furthermore, lung adenocarcinoma in females has increased markedly over the past 40 years due to smoking [5,6]. Compared to NSCLC, SCLC develops more aggressively, with earlier metastasis to organs, including brain, liver, adrenal glands, and bones, and a higher level of proliferation; it accounts for ~15% of all lung cancer cases [7,8]. Only one-third of patients with SCLC are diagnosed at an early stage [9]. Different treatments and therapies are used depending on the type of lung cancer. However, according to Cancer Research Institute, lung cancers are diagnosed at a late stage when monotherapies like surgery, chemotherapy, or radiation are often not effective and may even result in excessive healthy cell damage, e.g., in polychemotherapy. In this regard, new treatment methods, specifically immunotherapy, may be more relevant for patients with lung cancer, especially if combined with other modalities [10]. Previously, immunotherapy has not been as successful in treating lung cancer, leading to the common belief that lung cancer is not immunogenic [11]. However, lung cancer cells can evade the immune system in many ways, including secretion of immunosuppressive cytokines, loss of major histocompatibility complex antigen expression, and expression of molecules inhibiting T-cell activation. To augment the outcome of immunotherapy, various methods can be combined with the immunotherapy, e.g., hormonal therapy, chemotherapy, surgery, and radiation therapy. To highlight the applicability and effectiveness of the cell-based immunotherapy combined with another modality, we present the clinical case of a patient who was successfully treated by autologous activated lymphocytes combined with radiation therapy to deteriorate squamous cell carcinoma with metastases into the lymph node.

This clinical case is part of a research project conducted in accordance with the Declaration of Helsinki and approved by the Local Ethics Committee of the JSC National Scientific Medical Center (protocol 083/KI-77 on 3 August 2022). Informed consent was obtained from all subjects involved in the study. Written informed consent was obtained from the patient for publication of this paper and all images, clinical data, and other data included in the manuscript. The patient was informed about the progression of the condition, the possible outcomes if left untreated, and the possible complications of the combined treatment. The patient agreed to the proposed additional diagnostic tests and treatments, was satisfied with all procedures and manipulations, and had no treatment-related complications. The patient did not report any complaints regarding the financial costs associated with treatment. The manuscript was prepared according to the CARE guidelines [12].

## 2. Case Presentation

A 63-year-old man presented with persistent cough accompanied by difficulty in expectorating sputum and breathing deficiency while walking or climbing stairs, resulting in a persistent lack of physical activity. Chest computed tomography (CT, July and October 2023) revealed thickening of the walls of the left main bronchus and segmental bronchi on the left side. Bronchoscopy revealed infiltration in the left main bronchus. The patient underwent diagnostic left thoracotomy with biopsy of the mediastinal lymph nodes. Histological examination revealed squamous cell carcinoma metastasis with keratinization of the lymph nodes. Immunohistochemical examination revealed the positive expression of the P63 marker.

The patient was assigned to four courses of chemotherapy using the cisplatin + gemcitabine regimen for 3 months (from November 2023 to March 2024). Based on a control CT scan of the chest organs, no infiltrative or focal changes were detected. Lymphadenopathy was detected in bifurcated lymph nodes (up to 15 mm in size).

**Figure 1:**
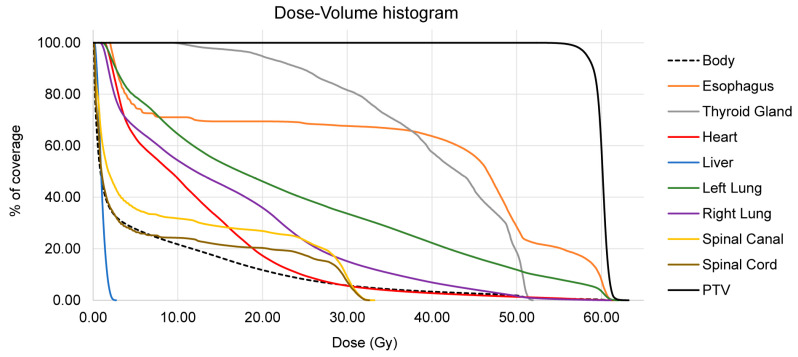
Dose–Volume histogram showing the relative dose coverage for key organs in the selected radiation therapy plan.

Due to the insufficient effect of mono-chemotherapy, the patient was recommended to undergo a combined treatment with immunotherapy using autologous activated lymphocytes and tomotherapy (spiral proton beam delivery with dose modulation under CT control); this treatment was performed from May to June 2024. Prior to the course of tomotherapy, pre-radiation topometric preparation was performed as follows: (i) native CT simulation on a 16-slice CT device (United Imaging, Shanghai, China) with the production of an individual fixation mask, (ii) selection of an individual regimen and volume of radiotherapy, (iii) individual dosimetric planning on TomoPlanning, and (iv) verification/dosimetric quality control of the treatment plan. Tomotherapy was carried out on the Radixact X9 device (Accuray, Madison, WI, USA) in TomoHelical mode from the area of the left lung formation (ROD-2 Gy, SOD-60 Gy) to the lymph nodes of the mediastinum (ROD-2 Gy, SOD-50 Gy). The treatment was 5 times a week, with daily visual control by image-guided radiation therapy, during which the precise guidance of the rays to the tumor area was checked using a megavoltage CT built into the TomoTherapy device. The dose–volume histogram for key organs in the selected regime of the radiation therapy plan is shown in Figure 1. The total duration of the tomotherapy course was 25 days, 5 weeks (except weekends). The patient received outpatient treatment.

### 2.1. Preparation of Cytokine-Activated Autologous Lymphocytes

To isolate and activate the lymphocytes, peripheral blood mononuclear cells from the patient (10 mL) were collected using an Optia apparatus (Terumo Penpol Pvt. Ltd., Thiruvananthapuram, India). A fraction of the lymphocytes was obtained from the collected biomaterial for further culturing. For culturing, we used Dulbecco’s modified Eagle’s medium (DMEM) and added interleukin (IL)-15, IL-7, FMS-like tyrosine kinase 3 ligand (Flt-3L), and stem cell factor (SCF) to the culture medium. During culturing and replacement of the nutrient medium, IL-2 was also used (to facilitate the growth and proliferation of T cells), as well as IL-15 and IL-7 (to promote the activation and proliferation of T lymphocytes and natural killer). The cell suspension was tested for Mycoplasma before administration to the patient.

**Figure 2:**
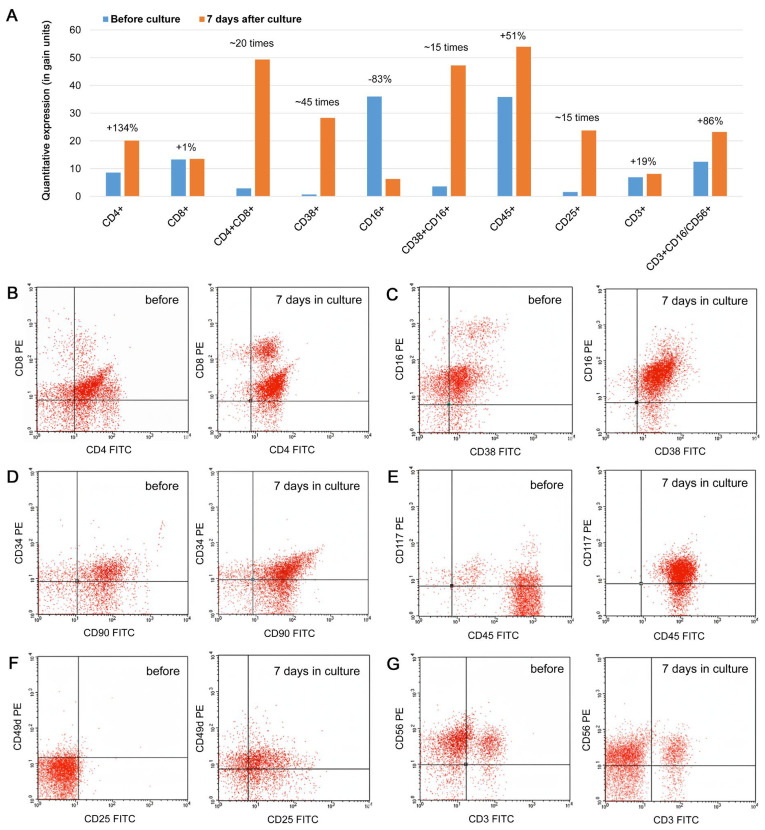
Comparison of surface marker expression in lymphocytes obtained from the patient and screened before culturing and 7 days after culturing in cytokine-containing media. (**A**) Quantitative gain measurements of various surface markers expressed by the lymphocytes before and after culturing (values are given as gain units). Percentages or rough quantitative changes are shown near to each pair of bars and reflect changes in 7 days culture compared to the measurement before culture. (**B**–**G**) Individual flow cytometry plots for the markers obtained before culturing the cells and 7 days after culturing the cells: (**B**) CD8 vs. CD4, (**C**) CD16 vs. CD38, (**D**) CD34 vs. CD90, (**E**) CD117 vs. CD45, (**F**) CD49d vs. CD25, (**G**) CD56 vs. CD3.

Various CD markers (for subsets of lymphocytes and for various activation states) were analyzed before and after cultivation of lymphocytes (Figure 2). After seven days of culture, the expression of the following markers was elevated: CD3+, CD3+CD8+, CD28+, CD29+, CD8+CD28+, CD38+, CD25+, CD45+, and CD3+CD16+/CD56+. Moreover, the proportion of double-phenotype T cells, CD4+CD8+, increased from 2.85 to 49.35 in gate units. In contrast, the expression of the markers CD16+, CD3+CD4+, and CD3-CD16+CD56+ (natural killer, NK) decreased. Cytotoxic CD8+CD28+ T lymphocytes and CD3+CD16+/CD56+ T lymphocytes increased by 67.1% and 86%, respectively, after 7 days of culture. Notably, among the entire population of T lymphocytes, CD3+CD16+/CD56+ cells are the most important effector cells that play a significant role in antitumor immunity. Thus, activation of these markers can be considered a positive sign of the activation of cellular immunity, which is important in assessing the immune status both in the infectious process and in tumors. Accordingly, these cells play a major role in eliminating tumor cells. The quantitative and percentage compositions of activated lymphocytes in cell culture were optimal for stimulating, maintaining, and enhancing antitumor immunity.

The purity of the cell culture for activated lymphocytes was around 85% (in proportion to non-lymphocytic cells) at the time of infusion. Activated lymphocytes were administered to the patient three times, each time fresh blood was collected to acquire peripheral blood mononuclear cells, and the administration was performed after 7 days of culturing. For the first administration, 1.86 × 10^7^ cells per 10 μL of the suspension was diluted in physiological solution (200 mL in total) for autogenous administration. For the second and third administrations, 7.36 × 10^6^ and 7.59 × 10^6^ cells per 10 μL, respectively, were prepared and each diluted in 200 mL of saline before infusion. Therefore, the patient received in each administration approximately the amount of cell as indicated above while we did not perform cell counting or concentration measurements of the diluted cell suspension. The interval between two consecutive administrations was 7–8 days to match the complete administration period (approximately 24 days) with the 25-day tomotherapy period. The patient’s health remained satisfactory during all treatments.

### 2.2. Circulating Tumor Cells as a Marker of Treatment Efficiency

The number of circulating tumor cells (CTCs) was assessed using flow cytometry. Briefly, 10 mL venous blood was collected from each patient. Leukocytes were isolated using the density gradient method. The cells were then stained with antibodies (EpCAM cell adhesion molecule (Alexa Fluor 488) and EGFR-PE endothelial growth factor), followed by washing off the unbound antibodies and performing flow cytometry (Attune NxT, Thermo Fisher Scientific, Waltham, MA, USA). CTCs were counted immediately before and three months after the end of the combined therapy.

To compare the measured CTC in the patients with a normal condition, we determined the CTC in the group with healthy individuals (with no pathology detected, *n* = 5) to obtain a reference level. The mean age of the volunteers was 35.6 years (28–48 years), and all the volunteers were women. The referent “normal” level was equal to 29.1 cells per million. Next, the number of CTC with the EGFR+EpCAM+ phenotype was determined in our patient before treatment, amounting to 30.8 cells per million. Three months later, i.e., after the completion of the treatment, this CTC level decreased to 28.4 CTC per million cells, so it was below both the pre-treatment level and the referent “normal” level.

### 2.3. CT Imaging

**Figure 3:**
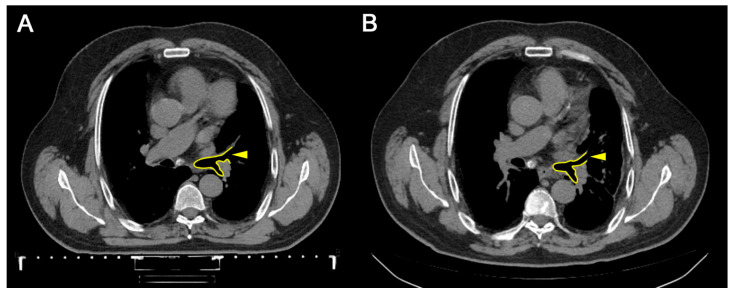
Computed tomography of the chest, axial section, the lumen of the left main bronchus. (**A**): Before the treatment by immunotherapy with autologous activated lymphocytes in combination with tomotherapy. (**B**): Three months after the treatment. Lumen is enclosed by yellow contour with a pointer; in dynamics, stabilization of the process is noted (size of the lumen is not changed).

**Figure 4:**
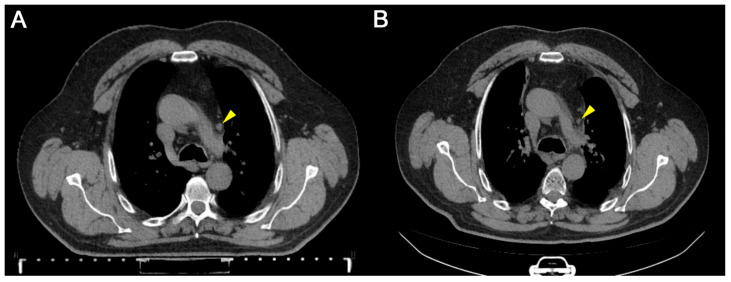
Computed tomography of the chest, axial section, the prevascular lymph node. (**A**): Before the treatment by immunotherapy with autologous activated lymphocytes in combination with tomotherapy. (**B**): Three months after the treatment. Lymph node is denoted by a yellow pointer; in dynamics, a substantial decrease in size of the lymph node is noted.

Three months later, after receiving a course of combined therapy, a CT scan revealed that the intrathoracic lymph nodes were slightly enlarged: the subcarinal was up to 1.5 cm in diameter and calcified; the paratracheal up to 0.6 cm, paresophageal up to 0.8 cm, and prevascular up to 0.5 cm (previously up to 0.9 cm). CT images of the chest before and after treatment are presented in Figure 3 and Figure 4, respectively. Compared to the CT obtained before immunotherapy in combination with tomotherapy, the patient showed improvements in the CT image, and there were stable dynamics without deterioration, with the exception of the appearance of areas of compaction in the parenchyma of the left lung. There was also a decrease in the size of the prevascular lymph node from 0.9 cm to 0.5 cm. CT results were consistent with the decreased concentrations of CTCs in the blood. During a survey by the attending physician, the patient noted an improvement in his well-being, a decrease in numbness in the lower extremities, an increase in sensitivity, an improvement in tolerance to targeted chemotherapy, an increase in endurance, and some reduction in shortness of breath during physical exertion.

## 3. Discussion

Our case of a 63-year-old patient with lung cancer shows that immunotherapy in combination with tomotherapy can be an effective treatment even in older patients. The patient’s health improved, the CTC level decreased after the treatment, and CT scans revealed stable dynamics without deterioration. A decrease in CTCs is an important criterion indicating a possible slowdown in the tumor process and a decrease in the risk of metastases, indicating the curative effect of the therapy.

Immunotherapy has revolutionized cancer treatment by improving the ability of the immune system to detect and fight cancer cells. Various approaches have been adopted to stimulate immune responses against cancer, including the use of therapeutic vaccines, immunomodulators, autologous cell therapy, and monoclonal antibodies. Some trigger the natural immune response of the body, whereas others activate cells to kill cancer cells. Immunotherapy can specifically target cancer cells that divide abnormally, while leaving healthy cells untouched. Autologous immune cell therapy utilizes cytotoxic lymphocytes, such as CD8+ and CD4+, which produce interferon gamma and play a significant role as tumor effector antagonists. If pre-activated, T lymphocytes can identify specific antigens displayed on the surface of tumor cells [13]. CD4+ T cells exert strong effector functions against infected or transformed cells; therefore, CD4+ cytotoxic cells are the most potent cells for immune cell-based immunotherapy [14].

Although TIL and CAR-T cell therapy are currently the most widely studied forms of personalized cellular immunotherapy, the use of cytokine-activated lymphocytes (PBMC-based therapy, including CIK/LAK cells) is also a scientifically sound approach and continues to be clinically relevant for several reasons. First, PBMCs activated by cytokines (IL-7, IL-15, SCF, Flt3-L) are a heterogeneous population of T cells with enhanced cytotoxic function. This provides an advantage in MHC-independent tumor cell recognition, which in turn allows for the destruction of tumors with low HLA expression, which possess mechanisms for immune evasion. Second, compared to TILs and CAR-T cells, the simplicity, availability, and speed of obtaining activated cells are advantageous. PBMC therapy does not require surgical harvesting of tumor tissue. This eliminates the need for lengthy TIL cell expansion or complex antigen specificity verification. Unlike CAR-T, there is no genetic modification step. This makes the method of injecting activated lymphocytes more accessible. This also makes the method suitable for patients with stage 3–4 cancer or for those unable to obtain sufficient TILs. Unlike TILs and CAR-T, it is a good option specifically for elderly patients or patients with comorbidities for whom aggressive TIL or CAR-T protocols may be contraindicated. Third, our reported case and unpublished cases of our other patients who received a similar therapy regimen for lung cancer demonstrated clinical efficacy: improved patient survival and demonstrated synergy with tomotherapy. The method of injecting activated lymphocytes provided clinical efficacy with low toxicity (which distinguishes it from some forms of CAR-T or intensive TIL protocols). In this reported case, the synergy of two therapies resulted in a clinical outcome because radiation therapy increases tumor immunogenicity, enhances the expression of stress-inducible molecules, and enhances immune cell infiltration. Fourth, activated lymphocytes have a high safety profile, a low risk of cytokine storm, minimal organ toxicity, and a good tolerability profile.

However, immunotherapy is not always effective and not successful in every patient with cancer. Patients who were unresponsive to immunotherapy had much higher levels of immature red blood cells, also known as CD71+ erythroid cells or circulating endothelial cells [15]. These cells have immunosuppressive properties that prevent the immune system from destroying cancer cells. Additionally, anemia, which is a common condition in patients with late-stage cancer, facilitates the elevated levels of circulating endothelial cells in those patients [15].

Also, a small number of patients have very high levels of PD-L1, a biomarker (or protein), indicating that the tumors are particularly susceptible to immunotherapy drugs. In patients with lung cancer, the proportion of those who express high enough levels of PD-L1 ranges from 23 to 86% [16]. Although higher levels of PD-L1 may be related to better outcomes of immunotherapy, it is not yet clear how PD-L1 levels can be used to plan treatment. Immunotherapy drugs may work in patients even if their PD-L1 levels are very low. To consider immunotherapy alone as the initial treatment for lung cancer, screening for PD-L1 levels is necessary. However, certain complications in lung cancer can be time-consuming: the ability to biopsy the tumor and assess the PD-L1 level depends on the location of the cancer cells; in some patients, it may not be possible to obtain a sufficient amount of tissue sample to determine the PD-L1 level [17]. Recently, it was found that while in tumors the antibodies bind to killer cells known as CD8+ T cells, in the lymph nodes, they interact much more with T follicular helper cells, a type of CD4+ T cell [18]. Follicular helper T cells do not attack the tumor directly; instead, they secrete IL-4, which stimulates T cell proliferation. Therefore, the effectiveness of immunotherapy can be explained by this key mechanism involving PD-1 antibodies.

Our study has some limitations. First, we did not perform PD-1/PD-L1 testing in this patient despite the combination of radiation therapy with chemotherapy, which is considered the standard of care for non-resectable NSCLC stage III, followed by immunotherapy with durvalumab, a PD-L1 inhibitor, to prevent recurrence. However, this treatment is extremely expensive, and at the national level in Kazakhstan, no cancer care program covers these costs. Therefore, treatment with a PD-L1 inhibitor imposes a substantial financial burden on the patient. Instead, we suggested concurrent radiation therapy and immunotherapy by activated autologous lymphocytes, which is not a standard treatment but is considered an alternative option in this specific case, particularly because it is cost-free for the patients (covered by the grant). The obtained effects—shrinkage of a prevascular lymph node, unchanged stable dimension of the lumen in the left main bronchus, and decreased number of CTC—support the validity of this combined treatment.

Second, we were unable to provide diagnostic follow-up for the patients because of their distant home locations. Therefore, no dynamic detection of CTC or CT imaging has been performed since the end of therapy. However, the patient is still alive with no reported deterioration or discomfort as surveyed by phone at the time of writing this case report (August 2025).

Third, in this specific case, we did not perform a single treatment (e.g., radiation therapy) as the initial stage of continuing therapy after fault in chemotherapy. It may be difficult therefore to distinguish between the effects of radiation therapy and immunotherapy. Considering the stage of the disease and the age of the patient, our multidisciplinary team recommended combined treatment without testing single variants. We also would like to note that this patient was among 10 other patients, which received a combination of cell immunotherapy and tomotherapy (main group). Treatment results for this group of patients demonstrated increased survival compared to the control group (10 patients without cell therapy, tomotherapy alone). In most cases, the main group demonstrated more pronounced decrease in tumor volume. The enhancement of the therapeutic effect demonstrates a clear temporal relationship with the administration of activated PBMCs. Despite limited tumor stabilization prior to cell therapy, the most pronounced regression or slowing of progression was observed after several cycles of PBMCs administration. Meanwhile, periods without cell therapy were characterized by less pronounced clinical dynamics. Patients also reported improved well-being during cell therapy. This pattern of observations forms a consistent causal pattern that meets key Bradford-Hill criteria, including temporal dependence, consistency, and biological plausibility, thereby confirming the significant contribution of PBMCs to achieving the observed therapeutic effect.

The observed clinical dynamics exceed those typical of isolated radiation therapy in both the severity and sustainability of the effect, confirming the contribution of PBMCs to the antitumor response. Thus, we attribute the enhanced therapeutic effect to the action of cytokine-activated PBMCs, as the combined clinical and biological data demonstrate a causal relationship between their administration and the observed improvement. The combination of biological plausibility, objective immunological data, a clear temporal relationship, and clinical observations provides compelling evidence that the patient’s improvement is due to the action of the administered cytokine-activated peripheral blood mononuclear cells.

The treatment by autologous pre-activated immune cells may induce adverse effects and it is therefore why it should be used in caution. The most common adverse events following infusion are low-grade fever, chills, headache, and fatigue [19]. Regarding safety of the treatment, the main additional risk with the use of cytokine-activated cells was an increased incidence of fever, while the risk of hematotoxicity, gastrointestinal toxicity, or liver injury was lower compared with chemotherapy alone. Specifically, following the infusion of cytokine-activated cells, most patients experienced only a mild fever, which was resolved within a few days without serious complications [20]. Also, a polyclonal population of CD3+CD56+ T cells with NK-like properties (CIK cells) have been successfully used in the treatment of a variety of solid tumors (including lung cancer, hepatocellular carcinoma, renal cell carcinoma, and lymphoma) with minor to moderate, manageable side effects. In addition, no cases of severe toxicity (grades III–IV), anaphylactic reactions, significant organ toxicity, or graft-versus-host disease were reported in the studies, highlighting the relatively favorable safety profile of autologous activated lymphocyte therapy [21]. CIK cell therapy is well tolerated in most patients with various types of cancer. The most common side effects are mild and transient (fever, fatigue, chills, mild infusion reactions). Severe or life-threatening complications are rare. Evidence of severe events directly related to CIK therapy (capillary leak syndrome, severe hypotension, prolonged cytopenia, autoimmune toxicity, increased risk of infections) is limited or absent, and long-term safety data are relatively scarce [19].

Many studies used combined activated lymphocyte therapy with chemotherapy or other treatments, making it difficult to directly attribute adverse events to activated lymphocytes. A number of publications use only descriptive safety indicators, without grading toxicity. The heterogeneity of cancer types, treatment regimens, lymphocyte activation protocols, and patient characteristics limits the ability to generalize the risks, especially in elderly patients. In our specific case, no adverse events were reported. On the other hand, more data need to be accumulated about the safety and effectiveness of the combined use of T cell-targeted immunotherapy with other anticancer treatments, in particular in elderly patients with late stages of the cancer.

## 4. Conclusions

Autologous activated lymphocyte immunotherapy in combination with radiation therapy may be an effective treatment for advanced-stage lung cancer in selected patients. Considering the age of our patient, it can be concluded that combined immunotherapy and tomotherapy may be considered a feasible treatment option for elderly patients as well.

## Data Availability

The research data were not published in publicly available repositories because all relevant data are included to the manuscript.

## Abbreviations

The following abbreviations are used in this manuscript:

CAR	Chimeric Antigen Receptor
CIK	Cytokine-Induced Killer
CT	Computed Tomography
CTC	Circulating Tumor Cells
EGFR	Epithelial Growth Factor Receptor
EpCAM	Epithelial cell adhesion molecule
LAK	Lymphocyte-Activated Killer
MHC	Major Histocompatibility Complex
NSCLC	Non-Small-Cell Lung Cancer
PD-1	Programmed Cell Death Protein 1
PD-L1	Ligand to Programmed Cell Death Protein 1
SCLC	Small-Cell Lung Cancer
TIL	Tumor-Infiltrating Lymphocyte
